# Efficacy of melatonin in alleviating disorders arising from repeated exposure to sevoflurane in males and females of the Wistar rats during preadolescence

**DOI:** 10.1038/s41598-024-62170-4

**Published:** 2024-05-24

**Authors:** Fatemeh Heydari, Mahdieh Nasiri, Arash Haroabadi, Javad Fahanik Babaei, Seyed Khalil Pestehei

**Affiliations:** 1 Department of Anesthesiology, Tehran University of Medical Sciences, Tehran, Iran; 2 Electrophysiology Research Center, Neuroscience Research Center Institute, Tehran University of Medical Sciences, Tehran, Iran; 3 Neuroscience Research Center Institute, Tehran University of Medical Sciences, Tehran, Iran

**Keywords:** Sevoflurane, Repeated exposure, Melatonin, Apoptosis, Neuroinflammation, Stress oxidative, Biochemistry, Biological techniques, Cell biology, Molecular biology, Neuroscience

## Abstract

Pediatricians use sevoflurane due to its fast action and short recovery time. However, studies have shown that repeated exposure to anesthesia can affect learning and memory. Melatonin, an indole-type neuroendocrine hormone, has significant anti-inflammatory, and neuroprotective properties. Melatonin’s impact on cognitive behavior in sevoflurane-anesthetized males and females of the Wistar rats during preadolescence was examined in this research. The cognitive function was evaluated by shuttle box and morris water maze tests, while interleukin-10, Catalase (CAT), Malondialdehyde (MDA), and Tumor Necrosis Factor-α (TNF-α) were evaluated using ELISA kits. The expression levels of the apoptosis-linked proteins, Bax, Bcl-2, and caspase-3, were determined using the western blotting technique. The learning and memory latencies of the rats were more significant in the sevoflurane groups than in the control group; however, the latencies were significantly shorter in the sevoflurane and melatonin groups than in the control group. The levels of MDA, TNF-α, Bax, and caspase-3 were significantly higher in the sevoflurane groups than in the control group. We also found that the levels of CAT and Bcl-2 were significantly reduced in the sevoflurane groups compared to the control group. Increasing levels of CAT, Bcl-2, and decreasing levels of MDA, TNF-α, Bax, and caspase-3 in response to melatonin indicate a possible contribution to the recovery from the sevoflurane impairment. Melatonin shows neuroprotective effects in male and female rats with sevoflurane-induced cognitive impairment. This suggests melatonin could be a valuable treatment for learning and memory deficits resulting from repeated exposure to sevoflurane, possibly by controlling apoptosis, oxidative stress, and inflammation.

## Introduction

Sevoflurane, a rapidly acting and low-solubility agent, is a popular choice among pediatric anesthesiologists for inhalational induction^1^. Despite its long-standing popularity, the impact of sevoflurane on short- and long-term neurological function remains unknown^2^. However, recent preclinical investigations have shed light on the potential harmful effects of sevoflurane on the developing brain. Animal studies have indicated that exposure to sevoflurane during critical development stages can lead to neuronal death and cognitive impairments^3^. Additionally, studies have demonstrated that sevoflurane, combined with hypoxia, can induce apoptosis in cultured nerve cells and increase the formation and accumulation of beta-amyloid protein^4,5^. Evidence also revealed that sevoflurane exposure can lead to apoptosis in brain tissue and result in behavioral disorders^6–8^.

Adolescents exposed to sevoflurane during brain development may experience learning and cognitive difficulties due to increased oxidative stress and reactive oxygen species (ROS) in the hippocampal nerve cells, leading to memory impairment^9^. Furthermore, neuroinflammation has been identified as a well-established sevoflurane-induced cognitive impairment pathway^10^. Results have shown that sevoflurane can elevate inflammatory markers such as IL-6, IL-10, and TNF-α levels in primary hippocampal neurons and blood samples^11,12^. Overall, these findings suggest that sevoflurane has the potential to induce neuroinflammation and cognitive impairment.

Women’s blood melatonin (MT) levels were found to be decreased when sevoflurane was used^13^. Melatonin (MT) is an indole-type neuroendocrine hormone with potent anti-inflammatory, anti-apoptotic, and neuroprotective properties^14,15^. MT has a favorable safety profile, and no significant side effects have been reported^16^. Through inhibition of cyclooxygenase-2 (COX-2) and nitric oxide synthase (NOS) enzymes, as well as activation of nuclear factor kappa-light-chain-enhancer of activated B cells (NF-Kβ), MT can act as an anti-inflammatory^17–19^. Additionally, MT can inhibit catalase, glutathione S-transferase, glutathione peroxidase, and glutathione reductase, all of which neutralize oxidative stress^20,21^. A study has shown that MT can ameliorate sevoflurane-induced cognitive dysfunction in aged mice^22^. Ample evidence suggests that MT can reduce oxidative stress, cell apoptosis, and inflammation.

The findings above provide substantial evidence regarding the detrimental impact of prolonged sevoflurane administration on apoptosis induction, ROS stress, and inflammation. In contrast to these effects, MT has been shown to mitigate oxidative stress, inhibit cell apoptosis, and attenuate inflammation. Consequently, MT administration may result in favorable outcomes in patients who require repeated sevoflurane anesthesia. These findings can offer medical practitioners a new approach to co-administer anesthetics and melatoninMTby mitigating the adverse effects of prolonged use (Fig. S1).

## Method and materials

### Animal

Pre-adolescent rats weighing 35–40 g were selected to begin sevoflurane exposure and treatment. This age was chosen based on previous literature outlining the timing of adolescence in rodents. Postnatal day (PND 25–30) marks the beginning of early adolescence in male and female rats, while PND 60–70 marks the beginning of early adulthood^23,24^. Seventy-two pre-adolescent male and female Wistar rats from eight mothers were randomly assigned to the experimental or control groups. The Tehran University of Medical Sciences experimental study center provided the mother rats. The rats were housed in four per cage at 22 ± 2 °C under 12-h light/dark cycle conditions. Throughout the study, animals were provided with standard food and water. The experiments were conducted between 9 A.M. and 4 P.M. This study was reported in accordance with ARRIVE guidelines. The laboratory animals were cared for and used following guidelines provided by the National Institutes of Health (NIH No: 8023, revised 1978). The Tehran University of Medical Sciences Ethics and Research Committee approved this experiment (IR TUMS.NI.REC.1400,05).

### Design of experiment

After weaning, the pre-adolescent animals were randomly divided into eight equal groups. These groups included a male and female control group, a sham + melatonin group (administrated melatonin at a dosage of 10 mg/kg daily for 15 days), a sevoflurane inhalation group (exposed to 2% daily for 15 days) and sevoflurane inhalation + melatonin group (exposed to 2% sevoflurane daily for 15 days along with melatonin at a dosage 10 mg/kg daily for 15 days). In the sevoflurane inhalation + melatonin group, melatonin was administered two hours after exposure to sevoflurane. Animals in the groups exposed to sevoflurane were initially given 8% for induction during the first minute of the exposure session. The concentration was then reduced to 2% with air/oxygen as a carrier at a gas flow rate of 2 L/min to maintain deep anesthesia Using a Bonther^®^ anesthesia apparatus (SP, Brazil), sevoflurane (Bremer Pharma GMBH, Germany). The loss of eyelid and righting reflexes confirmed deep anesthesia. During sevoflurane exposure, the chamber was heated to 38 °C. Upon moving freely, the rats were returned to their cages. The rats’ respiratory frequency and skin color were monitored throughout the anesthesia. After 15 days of anesthesia, the animals were cared for over 30 days before being prepared for the experiments.

### Studying behavioral

#### The Morris MWM water maze test

The Morris water maze is one of the most commonly used tasks in spatial learning and memory research. The maze used in this study was a circular black tank (120 cm diameter, 70 cm height) throughout the present study. Several visual cues were present in a dim room. A depth of 40 cm of tap water (22 ± 1 °C) was poured into the tank. The maze was divided into four equal quadrants, one contained an invisible circular platform (10 cm in diameter) submerged 1.5 cm beneath the water’s surface. Rats participating in the study were unable to see the platform. Fixed extra-maze visual cues surrounded the maze and remained constant throughout the test. The study consisted of two phases: acquisition and probe. Two weeks after surgery, the acquisition phases began. Rats were placed in the water in separate quadrants and allowed 90 s to locate the hidden platform. IF they did not find it within the time limit, they were guided to the platform and allowed to stay there for 30 s. A probe test was conducted 24 h after the final acquisition session and released into the water for 60 s to swim in the tank. The movement of animals was tracked automatically by a computerized system (EthoVision, Noldus, Version 11) using a charge-coupled device (CCD) camera positioned above the maze’s center. The latency to find the hidden platform (Escape latency) was analyzed in the acquisition phase. The probe tests measured the escape latency to the platform and the time spent in the target quadrant (time in the target zone).

#### Passive avoidance test

Passive avoidance apparatus has two compartments, one of which is brightly lit and the other dark. Through negative reinforcement, the animals are taught to avoid entering a dark chamber despite being punished. An illuminated chamber was connected to a dark chamber by a guillotine door. Electric shocks were delivered to the grid on the floor using an isolated stimulator. Each rat was placed into the apparatus for 10 min on the first day of testing to habituate to the apparatus. A trial of the acquisition was conducted on the second day. Rats were individually placed in the illuminated chamber. An inescapable electric shock (1 mA, 3 s once) was administered after the door was opened for habituation (10 min). The door was closed after entering the dark chamber, and an electric shock (1 mA, 3 s once) was delivered. Rats with an initial latency (IL) of more than 60 s were excluded from this study due to their excessive ILs. In the illuminated chamber, each rat was placed 24 h later. Step-through latency (STL) was measured between the illumination chamber and the dark chamber (up to a maximum of 600 s).

### Studying biochemical

Rats were sacrificed under ketamine anesthesia (150 mg/kg) after behavioral tests. The whole brain was isolated, cleaned, washed with ice-cold phosphate-buffered saline (PBS), hippocampus then isolated and homogenized in cold Tris–HCl buffer solution (150 mM, pH 7.4) with protease inhibitor. This assay was conducted after centrifuging the supernatant (1000 g, 4 °C, 10 min). Duplicate measurements were taken for all parameters. Total protein concentration was determined using the Bradford method^25^. Superoxide dismutase (SOD) activity was determined using a specific assay kit (Kiazist, LiveScience, Iran). Briefly, the supernatant was incubated with xanthine and xanthine oxidase in potassium phosphate buffer for 30 min, and nitroblue tetrazolium (NBT) was added. 550 nm was used to monitor the formation of blue formazan (n = 5 per group). Using an assay kit (Kiazist, Lifescience, Iran), we determined reduced glutathione (GSH) as an intracellular defensive element. In brief, 5% trichloroacetic acid was added to the supernatant, centrifuged, and then 0.1 ml of the obtained supernatant, 2 ml of phosphate buffer (pH 8.4), 0.5 ml of 5′5 dithio bis (2-nitrobenzoic acid) (DTNB), and 0.4 ml of distilled water were added. The absorbance was measured at 412 nm after 30 min of incubation^26^.

#### Oxidative stress and inflammatory measurement

The catalase enzyme neutralizes free radicals and reactive oxygen species in the body. Catalase converts hydrogen peroxide into water and oxygen molecules. Malondialdehyde (MDA), a marker of oxidative stress, is produced from polyunsaturated fatty acids. We used the measuring kit from the factory protocol (Catalase, MDA, Kiazist, LiveScience, Iran) to measure these substances. Additionally, cytokines IL-10 and TNF-α, known as anti-inflammatory and proinflammatory factors, were measured using relevant antibodies and kits with the ELISA method (Karmania Pars Gene, Iran) to assess inflammatory factors.

#### Apoptosis measurement through western blotting

The Western blot analysis was performed as previously described^27,28^. The tissue of hippocampus was lysed with RIPA buffer for western blotting. A centrifuge at 14,000 rpm for 20 min at 4 °C was used to remove the lysates. According to the manufacturer’s instructions, the Bradford Protein Quantification kit (DB0017, DNAbioTech, Iran) was used to determine protein concentration. A 2X Laemmli sample buffer was added to the tissue lysates. Lysates (20 μg) were then subjected to SDS-PAGE after 5 min boiling and transferred to a 0.2 μm immune-Blot™ polyvinylidene difluoride (PVDF) membrane (Cat No: 162-017777; Bio-Rad Laboratories, CA, USA). It was then blocked in 0.1% Tween 20 for 1 h with 5% BSA (Cat No: A-7888; Sigma Aldrich, MO, USA). The membranes were incubated for 1 h at room temperature with Anti-Bax (Cat No: ab32503, Abcam), Anti-Bcl2 (Cat No: ab194583, Abcam), and Anti-Caspase 3 (Cat No: ab184787, Abcam). Following TBST washing, membranes were incubated with goat anti-rabbit IgG H&L (HRP) (Cat No: ab6721; Abcam) secondary antibody. After that, the membranes were incubated for 1–2 min with enhanced chemiluminescence (ECL). We normalized protein expression to β-actin. Gel analyzer Version 2010a software (NIH, USA) was used to analyze protein bands by dividing the area under the curve for each band by the area under the curve for its corresponding actin band and comparing the calculated values between groups as previously described.

### Statistical analysis

The data was analyzed, and graphs were drawn using Graph Pad Prism software 8.0. Behavioral tests in groups were conducted using one-way ANOVA. We used one-way ANOVA to evaluate and compare biochemical and histological tests in groups and two pulses in groups. Additionally, *p* < 0.05 was considered significant, and if significant, Tukey’s post hoc test was used. Data are expressed as Mean ± SEM.

### Ethical approval

Ethics Approval All experiments were conducted according to the Guide for Care and Use of Laboratory Animals (National Institutes of Health Publication No. 80–23, revised 1996). Additionally, the Research and Ethics Committee of Tehran University of Medical Sciences ((IR TUMS.NI) reviewed and confirmed all procedures.REC.1400.05).

## Results

### Behavioral

#### The effect of melatonin on cognitive impairment caused by sevoflurane in male and female rats with the Morris water maze

**Figure 1:**
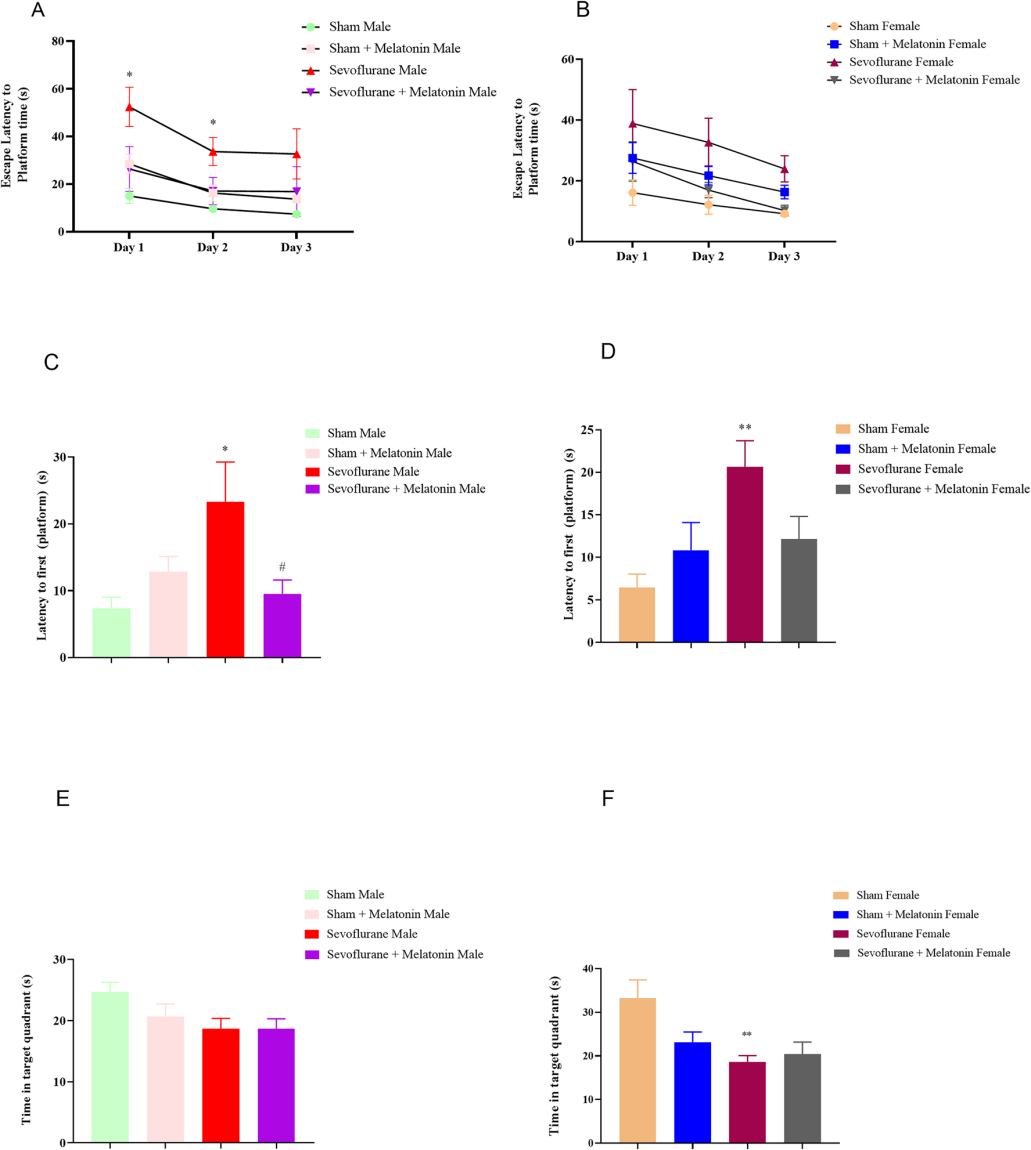
Test of Morris water maze (MWM) on the effect of melatonin on memory and learning in male and female rats exposed to sevoflurane repeatedly. (**A** & **B**) A two-way ANOVA of escape latency (the time to find the hidden platform, *p < 0.05). (**C** & **D**) Latency to the first platform (*p < 0.05, male sevoflurane compared to the male control group, #*p* < 0.05, male sevoflurane compared to the male sevoflurane and melatonin group, **p < 0.01 female sevoflurane compared to a female control group). (**E** & **F**) During the probe trial, sevoflurane-exposed female rats spent less time searching for the missing platform in the target quadrant than the controls, **p < 0.01). (**G**) Track the pathway showing time in the target zone by groups.

The MWM test was used to evaluate spatial learning and memory abilities in male and female rats exposed to sevoflurane and to assess the impact of melatonin consumption on these behaviors. Figure 1A,B show the escape latency to reach the platform during the acquisition phase of the MWM test. According to the repeated measures of a two-way ANOVA, all groups of rats showed gradual improvement in performance throughout the training period from days 1 to 3. Time trend analysis indicated that the latency to reach the platform during the learning phase significantly influenced both day factors [F _(1.94, 13.63)_: 13.08; *p* < 0.001 in males; F _(1.49, 8.98)_: 5.78; *p* < 0.05 in females] and group factors [F _(1.67, 11.75)_: 5.01; *p* < 0.05 in males; F _(1.45, 8.73)_: 5.18; *p* < 0.05 in females] but the interaction between the day and group did not have a significant impact [F _(2.46, 14.77)_: 0.68; *p* = *0.55* in male; F _(2.65, 14.59)_: 0.41; *p* = 0.71 in females]. Further analysis with One-way ANOVA revealed that animals in the male sevoflurane group had significantly longer latencies to reach the platform on days 1 and 2 (*p* < 0.05 on days 1 and 2) compared to the control male group and on day three compared to the female group (*p* < 0.05). However, there were no significant differences between males and females receiving sevoflurane compared to those receiving sevoflurane and melatonin. A probe trial test was conducted 24 h after the final training session to evaluate memory retrieval, with parameters including the latency to the first entry into the target (platform) and the total time spent in the target region. A significant effect of sevoflurane was observed on the first-entry latency [F _(3, 24)_: 4.04; *p* < 0.05 in males; F _(3, 26)_: 4.627; *p* = 0.01 in females]. Tukey’s *post-hoc* test confirmed that the sevoflurane male and female groups had higher latencies upon first entry to the target zone compared to the control male and female groups (*p* < 0.05 in the male groups and *p* < 0.01 in the female groups) (Fig. 1C,D). Additionally, the results indicated that melatonin reduced latency to the first entry to the target (platform) in males (but not females) compared to sevoflurane. There was a significant difference in the time spent in the target quadrant between the female sevoflurane group (but not the male group) and the female control group [F _(3,28)_: 5.189; *p* < 0.01] (Fig. 1E,F). Post hoc analyses showed that the female sevoflurane group spent less time in the target quadrant than the male control group (*p* < 0.05). Moreover, the results regarding the effect of melatonin on the total time spent in the target quadrant in male and female animals receiving sevoflurane showed no significant differences. The findings of this study suggest that repeated exposure to sevoflurane lead to memory impairment, which melatonin can improve. The track pathway comparison also supports these conclusions.

#### The effect of melatonin on passive avoidance behavior in male and female rats with repeated exposure to sevoflurane

**Figure 2:**
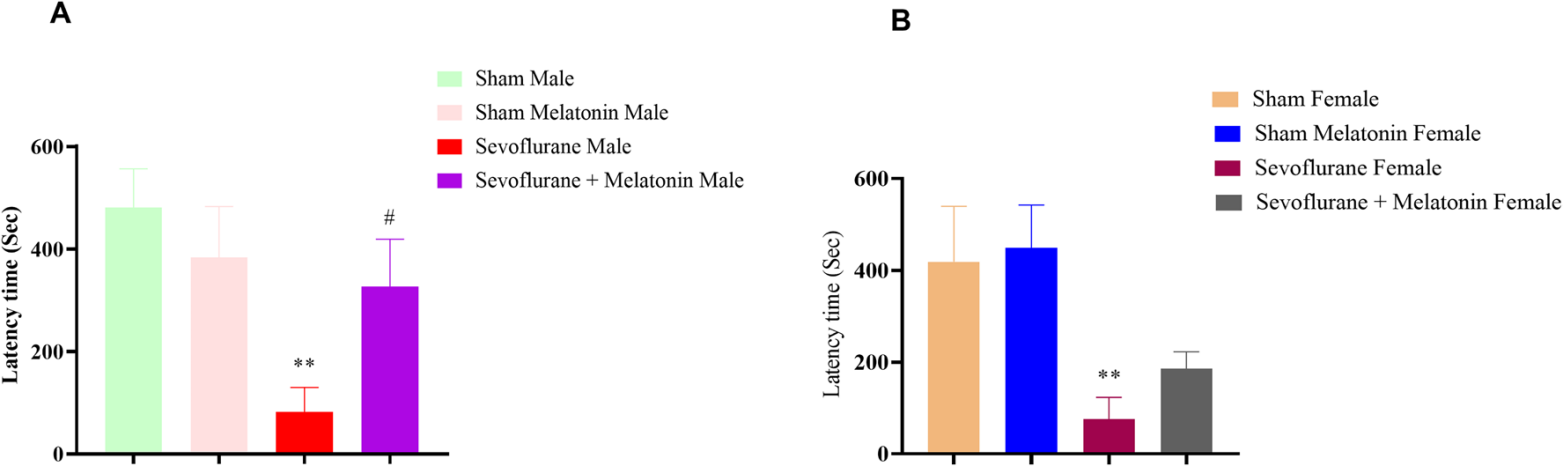
Passive avoidance behavior test on the effect of melatonin on memory and learning in male and female rats exposed to sevoflurane repeatedly. (**A**) A One-way ANOVA of latency time in males (**p < 0.01, male sevoflurane compared to a male control group and #*p* < 0.05, sevoflurane + melatonin male rats compared to male sevoflurane group. (**B**) A One-way ANOVA of latency time in females (**p < 0.01, sevoflurane-exposed female rats compared to control female group).

Passive avoidance learning to avoid certain stimuli, such as a particular object or situation, to prevent unpleasant or negative consequences. Our results indicated no significant differences in step-through latencies (STL) between groups during the training session (data not shown), making the groups homogeneous. However, the next day, the STL significantly decreased. Time spent in the dark compartment (TDC) increased in the sevoflurane male and female group relative to the control male and female groups (*p* < 0.01 in males [F (3,16): 4.39] and females [F (3,15): 9.30]). Interestingly, melatonin (sevoflurane + melatonin group) significantly increased STL (*p* < 0.05 in males but not females) compared to the sevoflurane group (Fig. 2A,B). Due to repeated sevoflurane exposure, melatonin treatment significantly improved retention test impairment. Our findings showed similar results when comparing the sham and sham and melatonin groups with the sevoflurane group.

### Biochemical

#### The effect of antioxidative melatonin on male and female rats repeatedly exposed to sevoflurane

**Figure 3:**
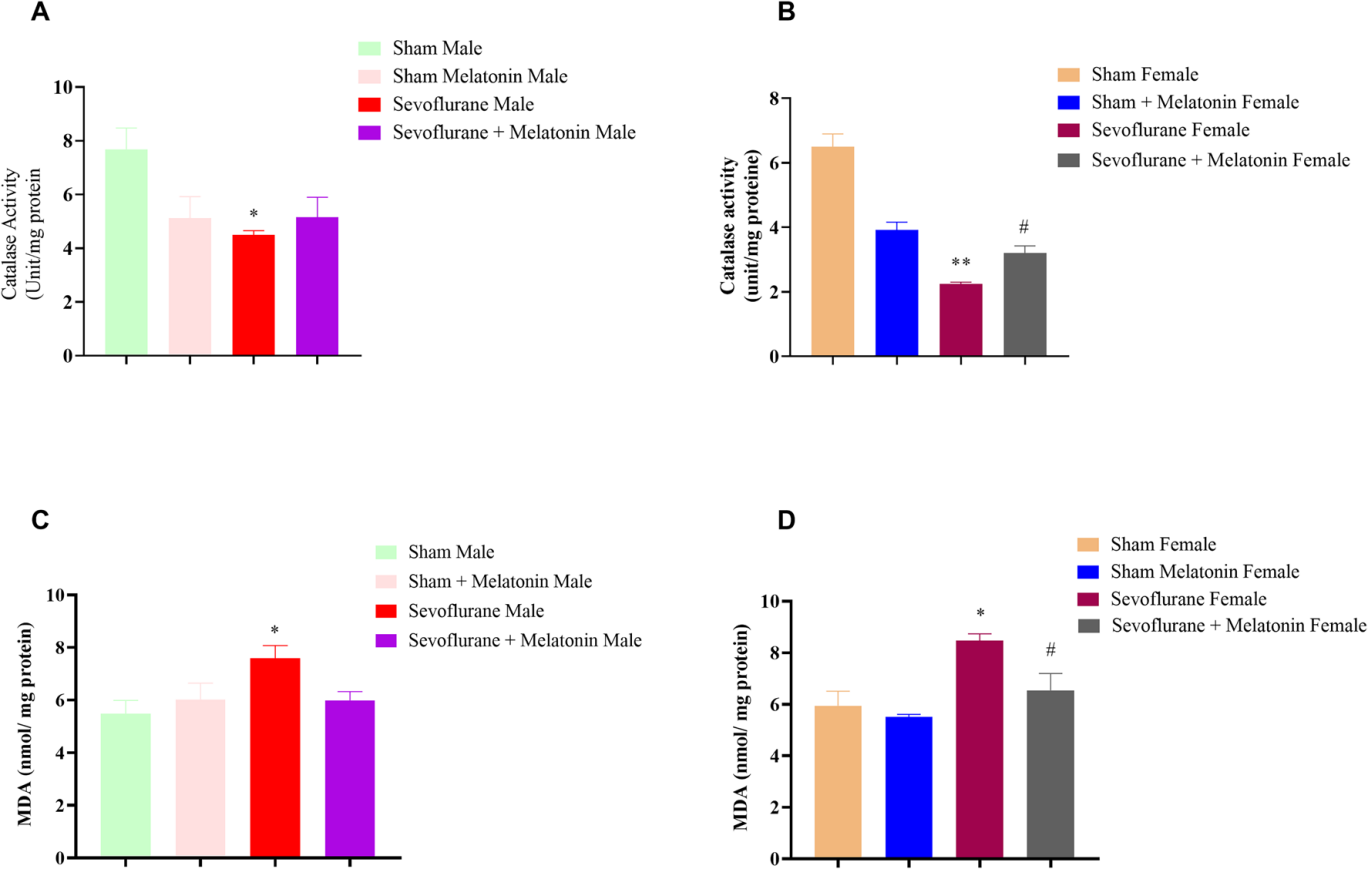
The effect of antioxidative melatonin on male and female rats repeatedly exposed to sevoflurane. (**A**) A One-way ANOVA of catalase activity in males (*p < 0.05, female sevoflurane compared to a female control group). (**B**) A One-way ANOVA of catalase activity in females (**p < 0.01, sevoflurane-exposed female rats compared to the control female group, and #*p* < 0.05, sevoflurane + melatonin group compared sevoflurane group). (**C**) A One-way ANOVA of the amount of MDA in female groups (*p < 0.05, sevoflurane male group compared to sevoflurane male group). (**D**) A One-way ANOVA of the amount of MDA in female groups (*p < 0.05, sevoflurane female group compared to sevoflurane female group, and #*p* < 0.05 sevoflurane + melatonin female group compared sevoflurane female group).

In addition to behavioral studies, two markers, CAT and MDA, were used to measure melatonin’s antioxidative effects. Sevoflurane significantly [F (4,5): 9.03; *p* < 0.05 in males; F (3,4): 14.43; *p* < 0.01 in females] reduced catalase activity in the hippocampus of adult males and females compared with the control group for oxidative stress markers in the hippocampus structure (Fig. 3A,B). Interestingly, melatonin only increased catalase enzyme activity in female animals (sevoflurane + melatonin) compared to the sevoflurane group [F (3,4): 7.90; *p* < 0.05] (Fig. 3B). One-way ANOVA showed a significant difference between groups in the increase of MDA in the hippocampus of male and female rats (Fig. 3C,D). Tukey’s post hoc test confirmed that the sevoflurane led to an increase in MDA compared to groups of control [F (4,12): 4.25; *p* < 0.05 in males; F (4,12): 5.52; *p* < 0.01 in females] and sham + melatonin male and female (*p* < 0.05), and melatonin decreased MDA only in the female sevoflurane + melatonin group compared sevoflurane group [F (4,12): 4.20; *p* < 0.05 in females]. Female rats administered repeated doses of sevoflurane showed reduced oxidative stress when treated with melatonin.

#### The effect of melatonin on inflammation markers in male and female rats with repeated exposure to sevoflurane

**Figure 4:**
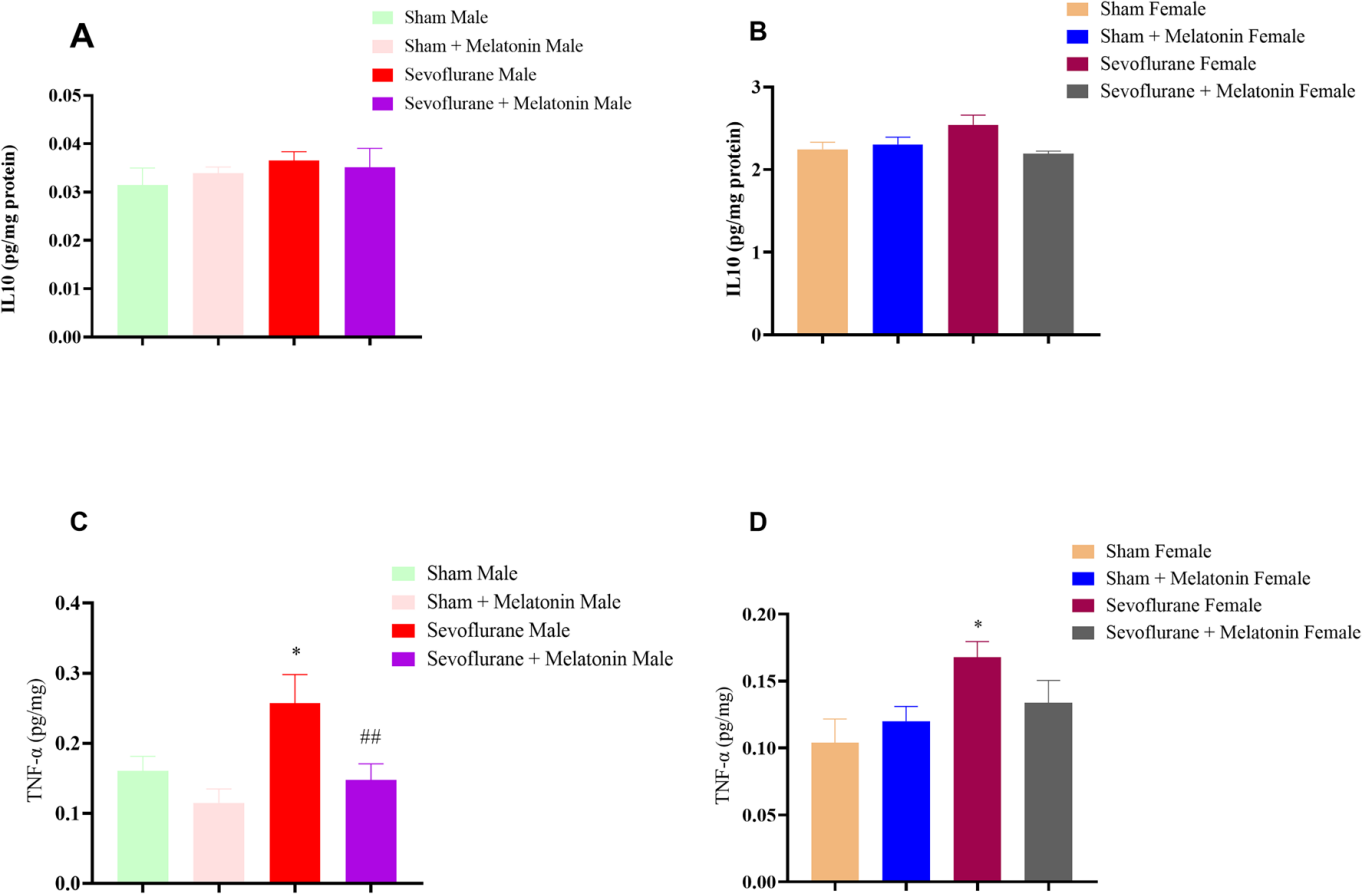
The effect of melatonin on inflammation markers in male and female rats with repeated exposure to sevoflurane. (**A** & **B**) One-way ANOVA analysis of measurement of IL-110 in male and female groups. (**C**) One-way ANOVA analysis of TNF-α in male groups (*p < 0.05, male sevoflurane compared to a male control group, ##*p* < 0.01, sevoflurane + melatonin male group compared male sevoflurane group). (**D**) One-way ANOVA analysis of TNF-α in female groups (*p < 0.05, sevoflurane + melatonin female group compared female sevoflurane group).

In the evolution of hippocampus inflammation factors (Fig. 4A–D), One-way ANOVA analysis indicated that sevoflurane led to increased inflammation factors compared to the control group. This increase in TNF-α was statistically significant [F (4,15): 5.34; *p* < 0.01 in males; F (4,15): 4.53; *p* < 0.05 in females] but IL-10 was not significant in the male group. Only the TNF-α factor in female rats showed a significant difference in inflammation compared to the sevoflurane group among the various inflammatory factors.

#### The effect of melatonin on apoptosis markers in male and female rats with repeated exposure to sevoflurane

**Figure 5:**
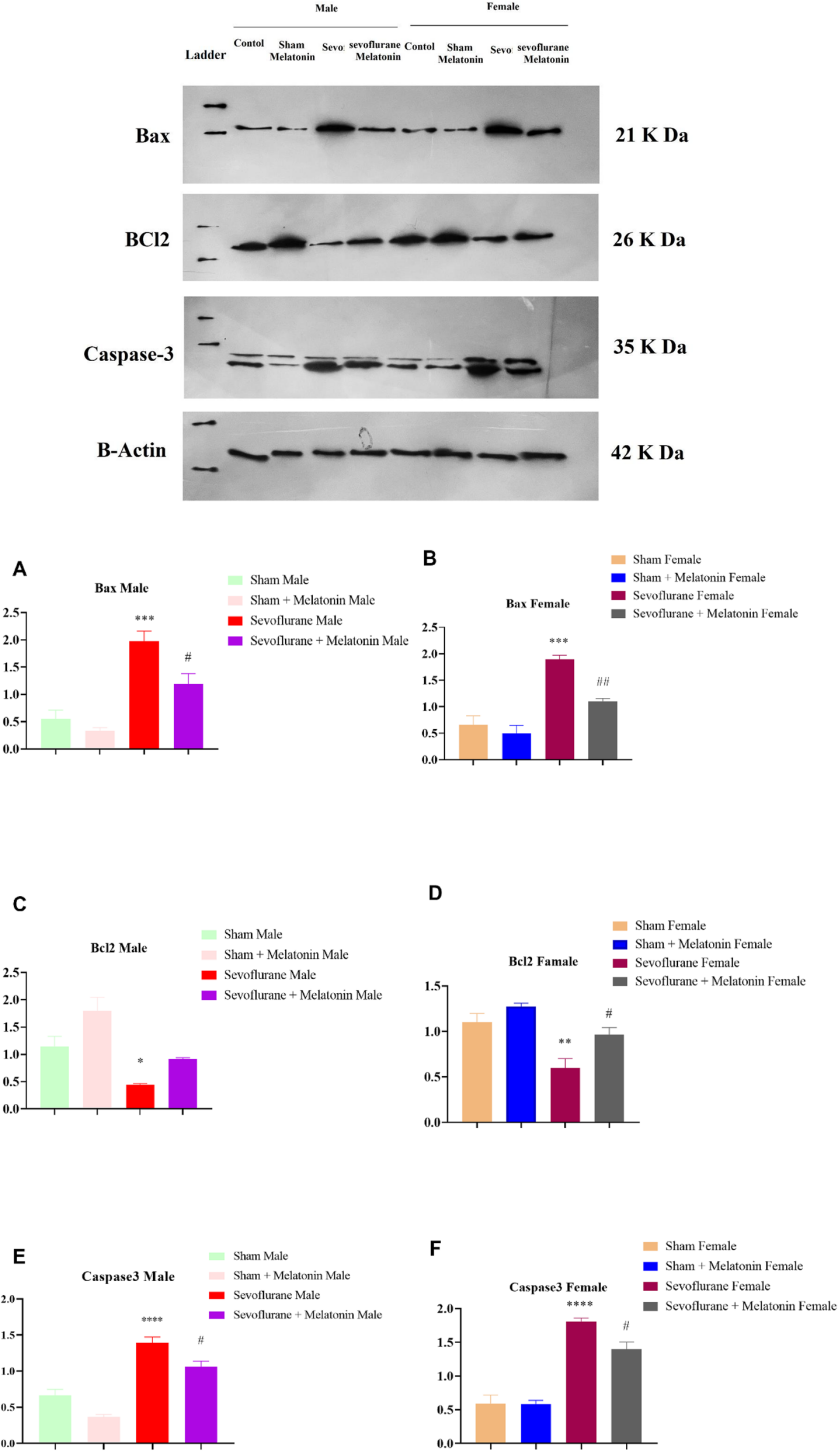
The effect of melatonin on apoptosis markers in male and female rats with repeated exposure to sevoflurane. (**A**) One-way ANOVA analysis of Bax in male groups (***p < 0.001, male sevoflurane compared to a male control group, and #*p* < 0.05, sevoflurane + melatonin male group compared male sevoflurane group). (**B**) One-way ANOVA analysis of Bax in female groups (***p < 0.001, female sevoflurane compared to a female control group, and ##*p* < 0.01, sevoflurane + melatonin female group compared female sevoflurane group). (**C**) One-way ANOVA analysis of Bcl2 in male groups (*p < 0.05, sevoflurane male group compared to male group). (**D**) One-way ANOVA analysis of Bcl2 in female groups (**p < 0.01, sevoflurane female group compared to female control group, #*p* < 0.05, sevoflurane + melatonin female group compared to female sevoflurane group). (**E**) One-way ANOVA analysis of caspase-3 in male groups (***p < 0.001, male sevoflurane compared to a male control group, and #*p* < 0.05, sevoflurane + melatonin male group compared to male sevoflurane group). (**F**) One-way ANOVA analysis of caspase-3 in female groups (***p < 0.001, female sevoflurane compared to a female control group, and #*p* < 0.05, sevoflurane + melatonin female group compared to female sevoflurane group).

The next step was to determine if the expression levels of key proteins in the apoptotic in hippocampus pathway could predict cellular response to treatment. The amounts of proapoptotic-related proteins (Bax, Caspase-3) [F (3,8): 21.7; *p* = 0.0003 in males; F (3,8): 26.29; *p* = 0.0002 in females (Bax), F (3,12): 35.72; *p* < 0.0001 in males; F (3,12): 44.99; *p* < 0.0001 in females (Caspase-3)] and antiapoptotic-related proteins (BCl2) 3 [F (3,12): 13.30; *p* = 0.0004 in males; F (3,12): 11.95; *p* = 0.0006 in females (BCl2] were determined. Based on the statistical analysis, we found that melatonin (sevoflurane and melatonin) treatment caused a decrease in Bax and caspase-3 protein levels in both males and females (Fig. 5A–F) and an increase in Bcl2 protein levels in females (but not males) compared to the sevoflurane group.

## Discussion

In the present study, rats in the sevoflurane group showed weakened memory compared to those in the control group, as indicated by the results of the MWM test. The shuttle box assay further confirmed the impairment of learning and memory caused by sevoflurane, which was consistent with the MWM test findings. These results demonstrate that repeated exposure to sevoflurane induces cognitive impairment in male and female preadolescent rats. From a mechanistic standpoint, sevoflurane significantly increased apoptosis, oxidative stress, and inflammation in both male and female rats. To address this, we utilized melatonin, known for its anti-inflammatory, anti-apoptotic, and neuroprotective properties, to mitigate cognitive dysfunction resulting from repeated exposure to sevoflurane during preadolescence. Our study findings suggest that melatonin effectively improves cognitive deficits by regulating apoptosis, inflammation, and oxidative stress responses in the brain.

Sevoflurane, a volatile anesthetic, and highly fluorinated methyl isopropyl ether, is the most popular choice for pediatric patients due to its quick onset and recovery^29^. The neurotoxic effects of sevoflurane may impair brain development and impact long-term learning and memory in animals^30–32^. Our study revealed that sevoflurane-induced learning and memory impairments were observed in male and female preadolescent rats, consistent with previous reports of hippocampal-dependent memory deficits from sevoflurane exposure. Exposure to sevoflurane in neonates has been shown to result in long-term cognitive damage, including memory loss and anxiety^33^. According to researchers in 2022, neonatal exposure to sevoflurane can impair cognition in adolescent rats by altering hippocampal metabolism^34^. Another study found that exposure to sevoflurane in childhood could impair spatial memory in adulthood, with greater doses, shorter exposure intervals, and younger age at exposure leading to worsened memory function in adults^35^. Multiple exposures to sevoflurane during postnatal development have been linked to cognitive function decline in older adults^36^. Sevoflurane-induced neurotoxicity may impact cognitive performance by disrupting brain glucose metabolism during development, as shown by reduced neuronal glucose transporter 3 (GLUT3) levels impairing learning and memory in young mice^37^. In adult mice, sevoflurane exposure results in impaired short-term memory due to decreased synaptic transmission caused by inhibition of postsynaptic density protein 95 (PSD-95) and AMPA receptors in the hippocampus^38^. Our findings align with previous research indicating that repeated exposure to sevoflurane can negatively affect memory and learning in both male and female rats.

Anesthesia with sevoflurane induces oxidative stress and cognitive impairment in hippocampal neurons of older rats^9^. Malondialdehyde (MDA) is a biomarker for oxidative stress produced in the body through lipid peroxidation. The current study compared male and female rats treated with sevoflurane to control groups, revealing MDA levels. Along with the increase in MDA levels, there was a reduction in the level of catalase enzyme, which is responsible for anti-oxidative damage. This increase in oxidative stress could lead to an increase in apoptosis. Additionally, increased levels of malondialdehyde (MDA), IL-17A, NF-κB p65, inducible nitric oxide synthase (iNOS), and COX-2, as well as a reduced level of superoxide dismutase (SOD), were also observed in sevoflurane anesthetized aged rats^29^. To understand neonatal anesthesia-induced oxidative stress, it is vital to observe the differences between the adult and neonatal antioxidant defense systems^30^. An animal model has shown that the young brain has low glutathione peroxidase activity and is sensitive to oxidative stress^31^. Another study found that overexpression of SOD1 in the newborn brain is harmful because downstream enzymes such as glutathione peroxidase and catalase do not compensate^32^. These findings support the idea that superoxide dismutase and glutathione peroxidase play a significant role in the neonatal antioxidant defense system, as indicated by numerous studies that have primarily utilized mimicking agents^33–36^. Human studies suggest that full-term newborns are sensitive to oxidative stress, which resolves with maturity^37^, representing a temporary imbalance in the ROS-antioxidative system. Therefore, oxidative stress is more likely to impact the newborn brain due to lower levels of antioxidants.

Prolonged exposure to sevoflurane may disrupt late-stage progenitor granule cell development in the hippocampal dentate gyrus (DG), leading to cognitive impairments through autophagy-activated apoptosis via NF-κB activation^38^. Research has also shown that long-term exposure to sevoflurane can damage neurons and impair cognition in aging rats, potentially due to sevoflurane-induced memory impairment associated with endoplasmic reticulum **(**ER) stress-mediated neuronal apoptosis^39^. Previous studies have indicated that sevoflurane increases caspase-3 expression^40,41^, also observed in our research. Our study additionally found a connection between caspase-3 and sevoflurane-induced memory impairment in rats, with increased caspase-3 expression and Poly (ADP-ribose) polymerase (PARP) cleavage in hippocampus tissue contributing to neuronal death and significant memory impairment^42,43^. This suggests that caspase-3-induced PARP cleavage may be a mechanism for hippocampal neuronal death and memory impairment in postnatal mice^44^.

The results of our study indicate that repeatedly administering sevoflurane led to an increase in TNF-α levels in both male and female rats. However, sevoflurane did not affect the levels of IL-10, known for its anti-inflammatory properties. Dysregulated neuroinflammation has been linked to cognitive dysfunction^22^. Qiao et al.^45^ reported that individuals undergoing sevoflurane anesthesia had higher plasma levels of S-100β protein, TNF-α, and IL-6. Yang et al.^46^ demonstrated that sevoflurane administration in older rats could stimulate inflammation and lead to neuronal death in the hippocampus. Multiple studies have shown a significant increase in pro-inflammatory cytokines IL-1β, IL-6, and TNF-α in the brains of rats exposed to sevoflurane^47,48^.

Our results indicate that melatonin can improve cognitive dysfunction (learning and memory) in male and female rats during preadolescence. This improvement is achieved through the regulation of oxidative stress (decreased MDA levels), anti-oxidant activity (increased CAT levels), inflammation (decreased TNF-α levels), and apoptosis (increased Bax, decreased Bcl-2 and increased caspase-3) response in the brain. Melatonin is a neuroendocrine hormone of the indole type that performs a wide range of biological activities. It exhibits anti-inflammatory, anti-apoptotic, anti-oxidant, and neuroprotective properties. Melatonin production peaks between 3 and 6 and decreases slightly throughout adolescence. After the age of 35, melatonin secretion significantly declines. Every 10 years, melatonin secretion decreases by 10–15%^49^. In cases of oxidative stress, melatonin plays a protective role for mitochondria. Its complex enzymatic process helps reduce oxidative damage^50^. Melatonin scavenges NO·, reduces NOS activity, acts as an indirect antioxidant, and promotes superoxide dismutase activity, converting O2^−^ to less harmful H_2_O_2_. Melatonin also stimulates catalase and glutathione peroxidase^51^. Anesthesia and surgery can affect melatonin secretion, and disrupting melatonin production can lead to anxiety and postoperative neurocognitive impairment^52^. A study has shown that melatonin significantly reduced cognitive dysfunction in aged mice induced by sevoflurane^22^. ER stress caused by sevoflurane has been linked to hippocampal damage^53^. Melatonin appears to protect against anesthesia-induced neurotoxicity associated with ER stress^54^. Further research has suggested that melatonin may improve spatial learning and memory deficits resulting from exposure to isoflurane by reducing ER stress, neuronal apoptosis, and neuroinflammation through the activation of the SIRT1/Mfn2/PERK signaling pathway^55^. Melatonin also restores normal Bax and Bcl-2 protein expression in the dentate gyrus subgranular zone of pinealectomized rats, leading to increased spatial learning and memory by reducing cellular death^56^.

## Conclusion and future perspective

During pre-adolescence, repeated exposure to sevoflurane can cause rapid changes in the hippocampus. The effects of acute brain injury persist into adulthood, resulting in cell apoptosis, inflammation, and oxidative stress. Our study found that melatonin mitigated sevoflurane-induced cognitive impairment in male and female rats during preadolescence. As a result, melatonin may be a valuable therapeutic option in addressing sevoflurane-induced learning and memory deficits by regulating apoptosis, inflammation, and oxidative stress.

### Supplementary Information

Supplementary Figures.

## Data Availability

Data and material are available to the corresponding authors upon request.
