# Supplementary material for: Efficacy of melatonin in alleviating disorders arising from repeated exposure to sevoflurane in males and females of the Wistar rats during preadolescence
Source: Sci Rep. 2024 May 24;14:11889. doi: 10.1038/s41598-024-62170-4 (PMC11126601; doi:10.1038/s41598-024-62170-4)
Supplement: Supplementary file 1 — Supplementary Figures. [file 41598_2024_62170_MOESM1_ESM.pdf]

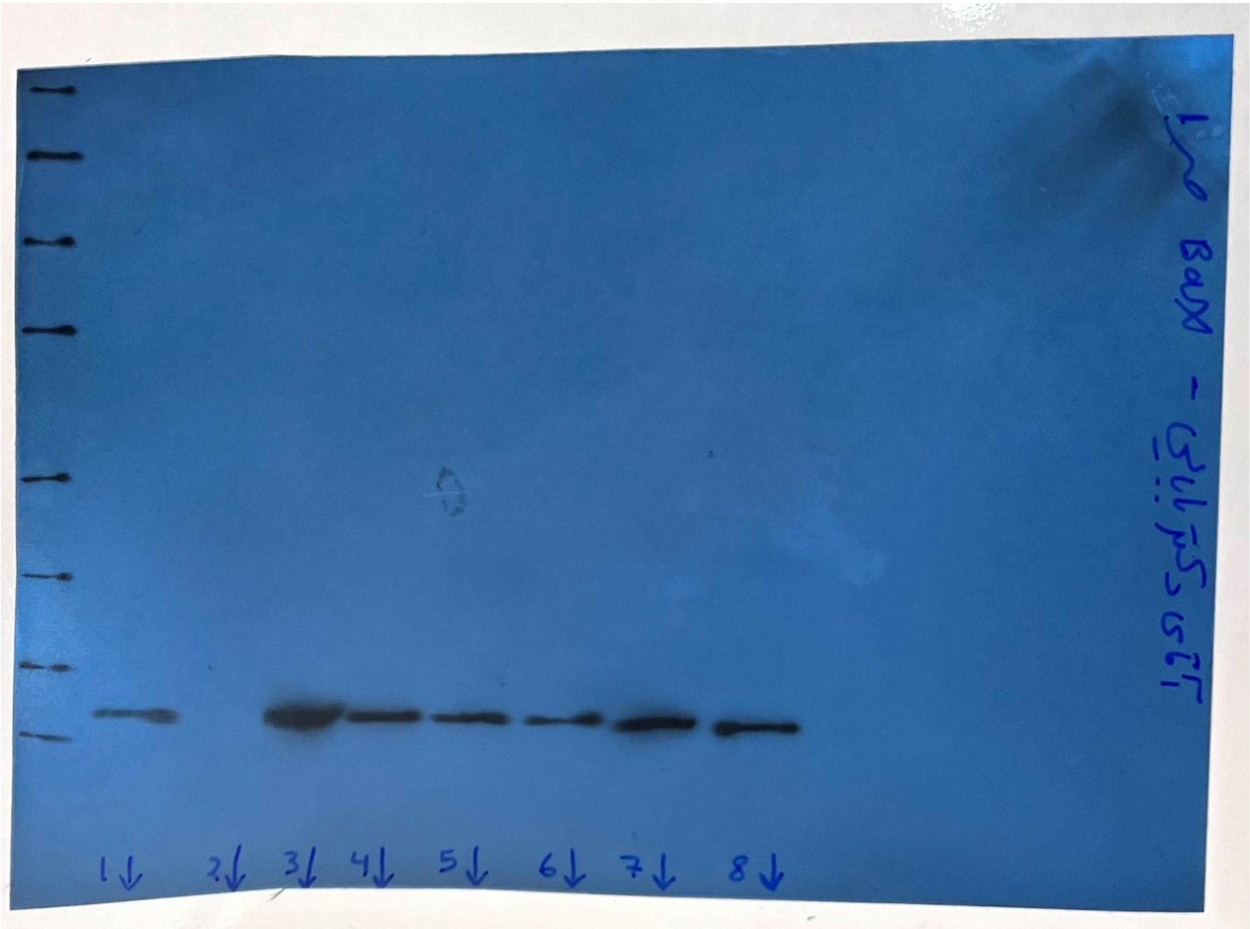

Fig S1: X-ray film of a Western blot showing Bax protein expression in hippocampal tissue (page 1).

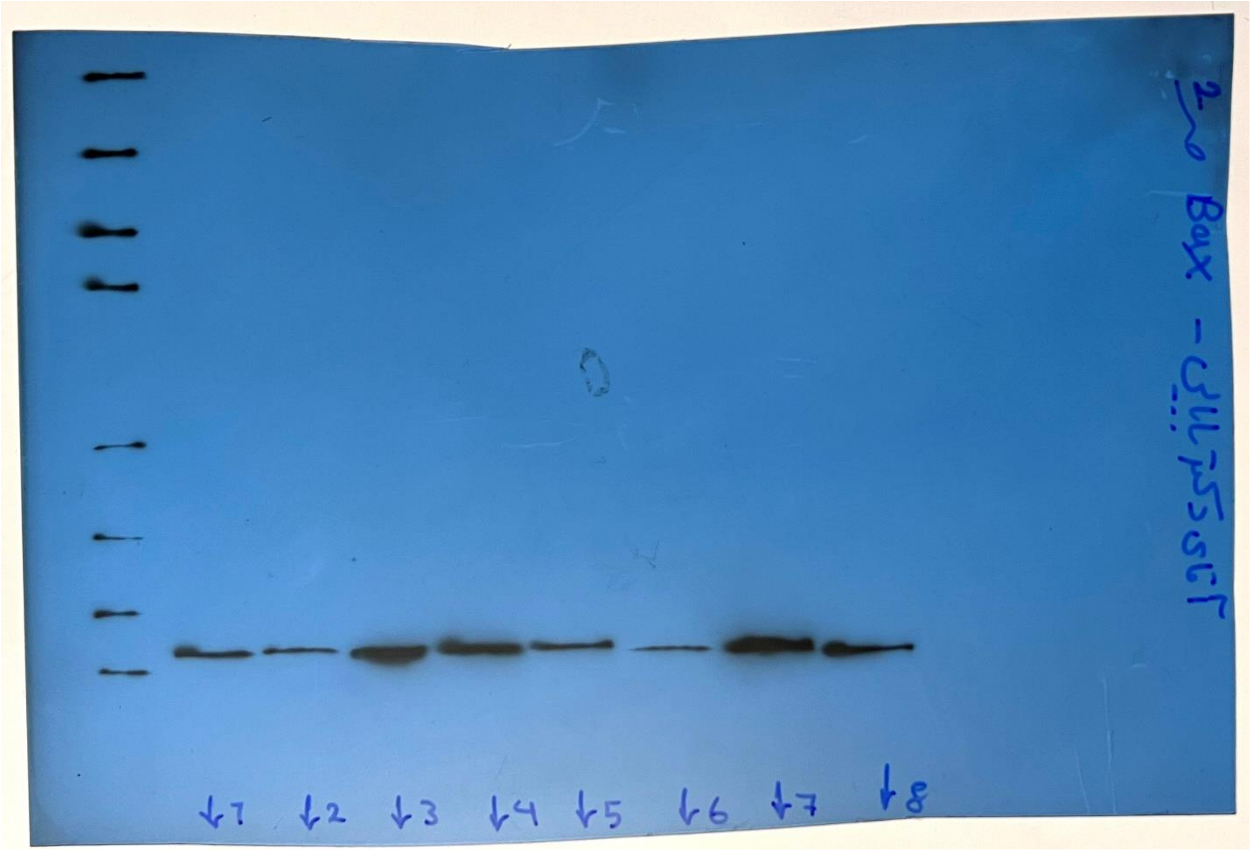

Fig S2: X-ray film of a Western blot showing Bax protein expression in hippocampal tissue (page 2).

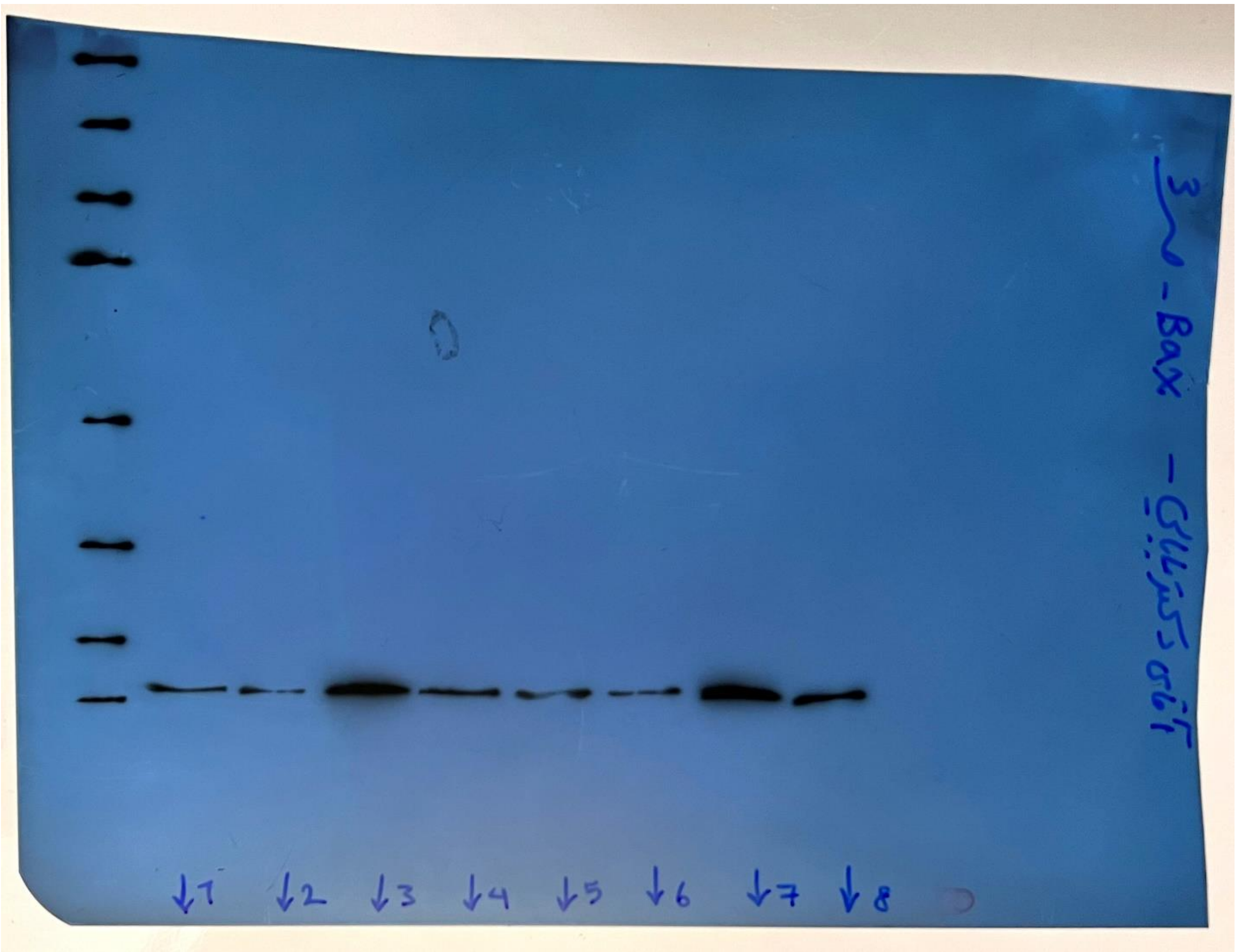

Fig S3: X-ray film of a Western blot showing Bax protein expression in hippocampal tissue (page 3).

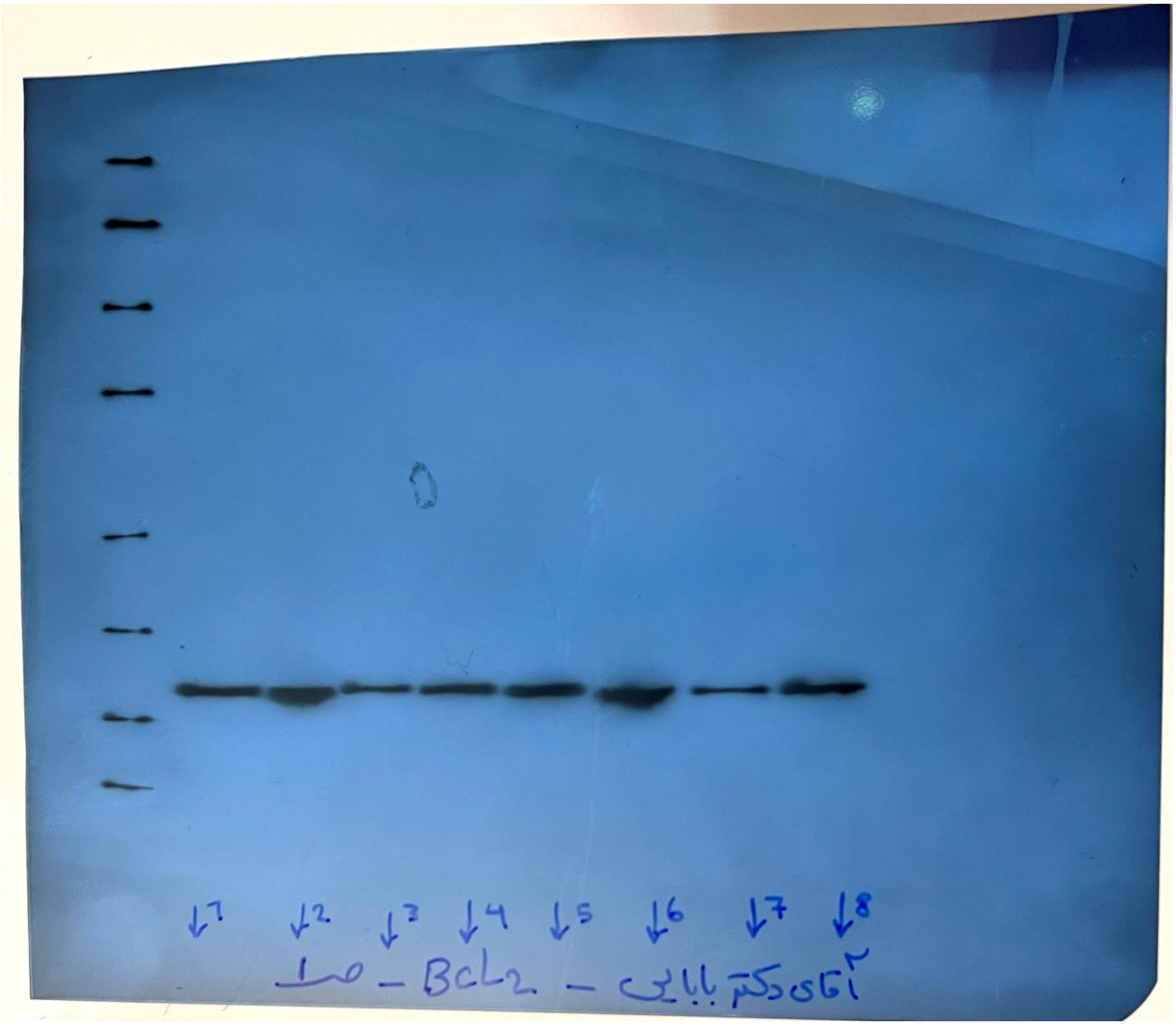

Fig S4: X-ray film of a Western blot showing Bcl-2 protein expression in hippocampal tissue (page 1).

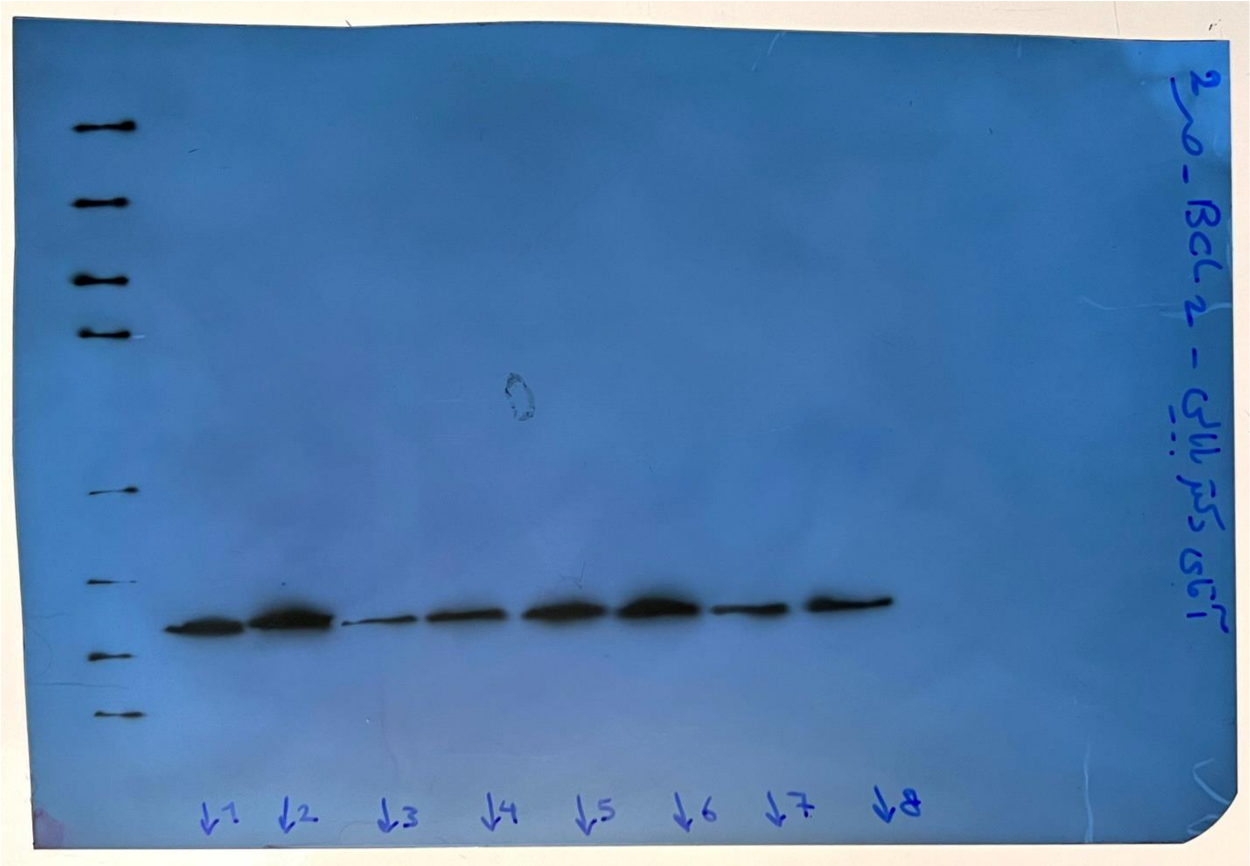

Fig S5: X-ray film of a Western blot showing Bcl-2 protein expression in hippocampal tissue (page 2).

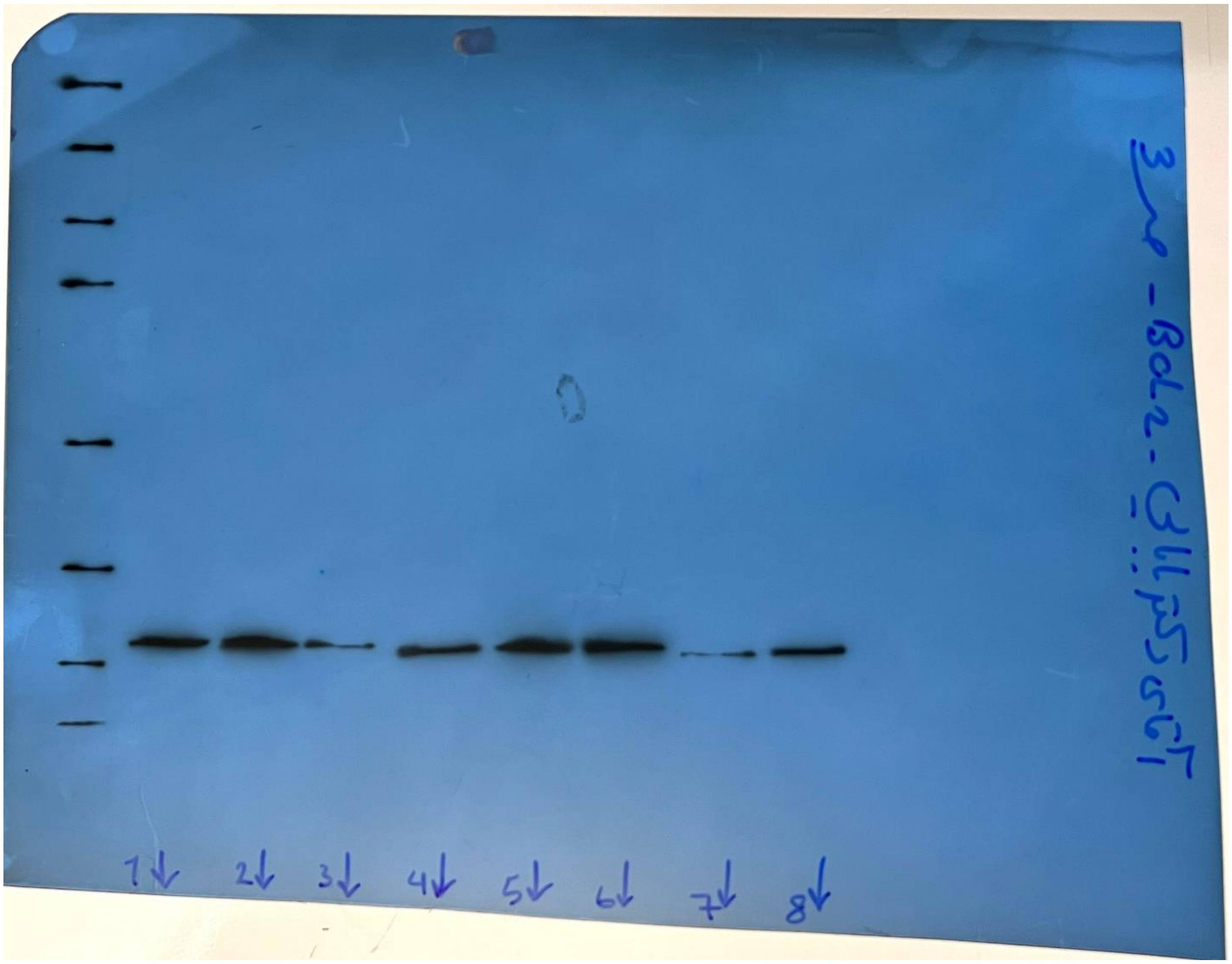

Fig S6: X-ray film of a Western blot showing Bcl-2 protein expression in hippocampal tissue (page 3).

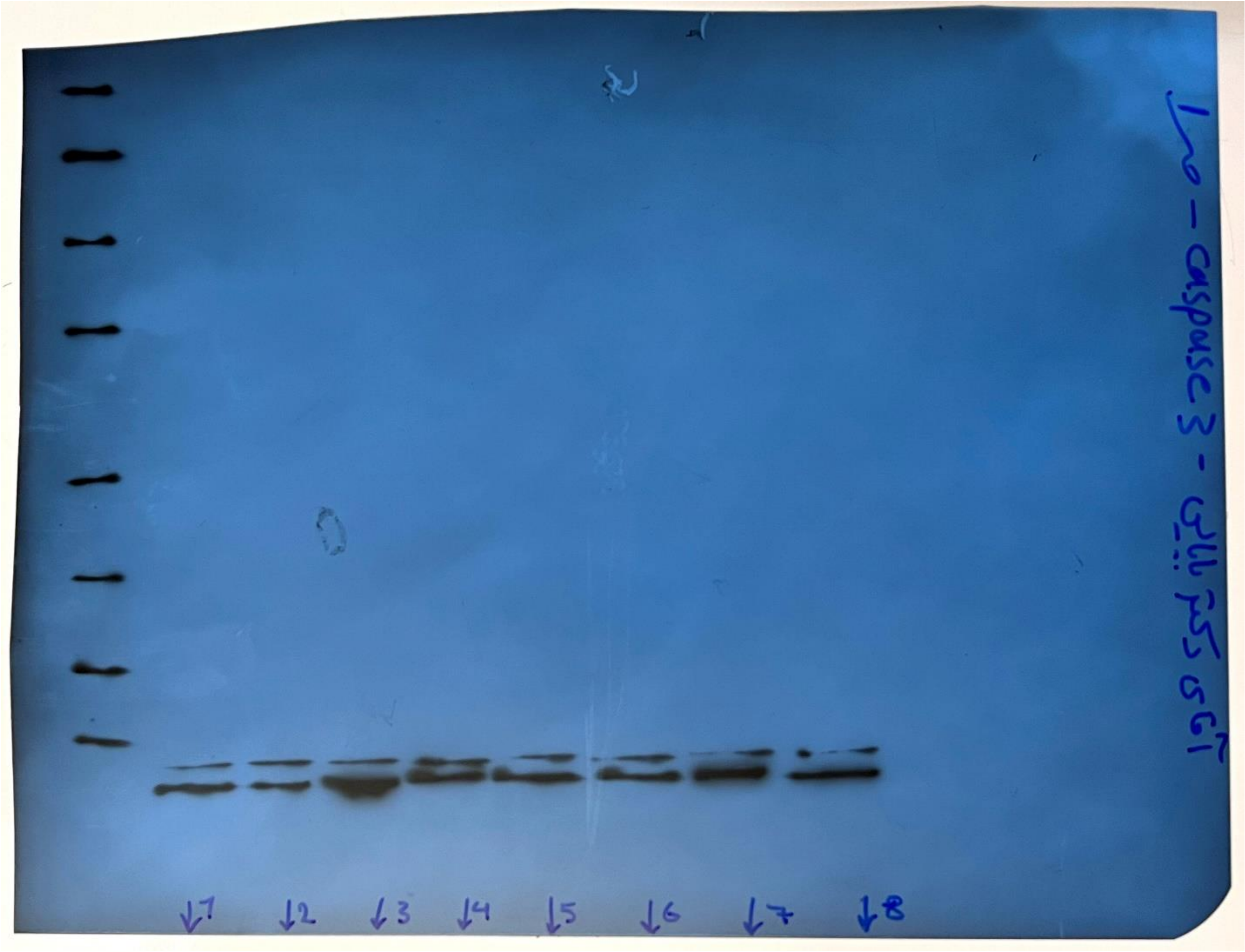

Fig S7: X-ray film of a Western blot showing Caspase-3 protein expression in hippocampal tissue (page 1).

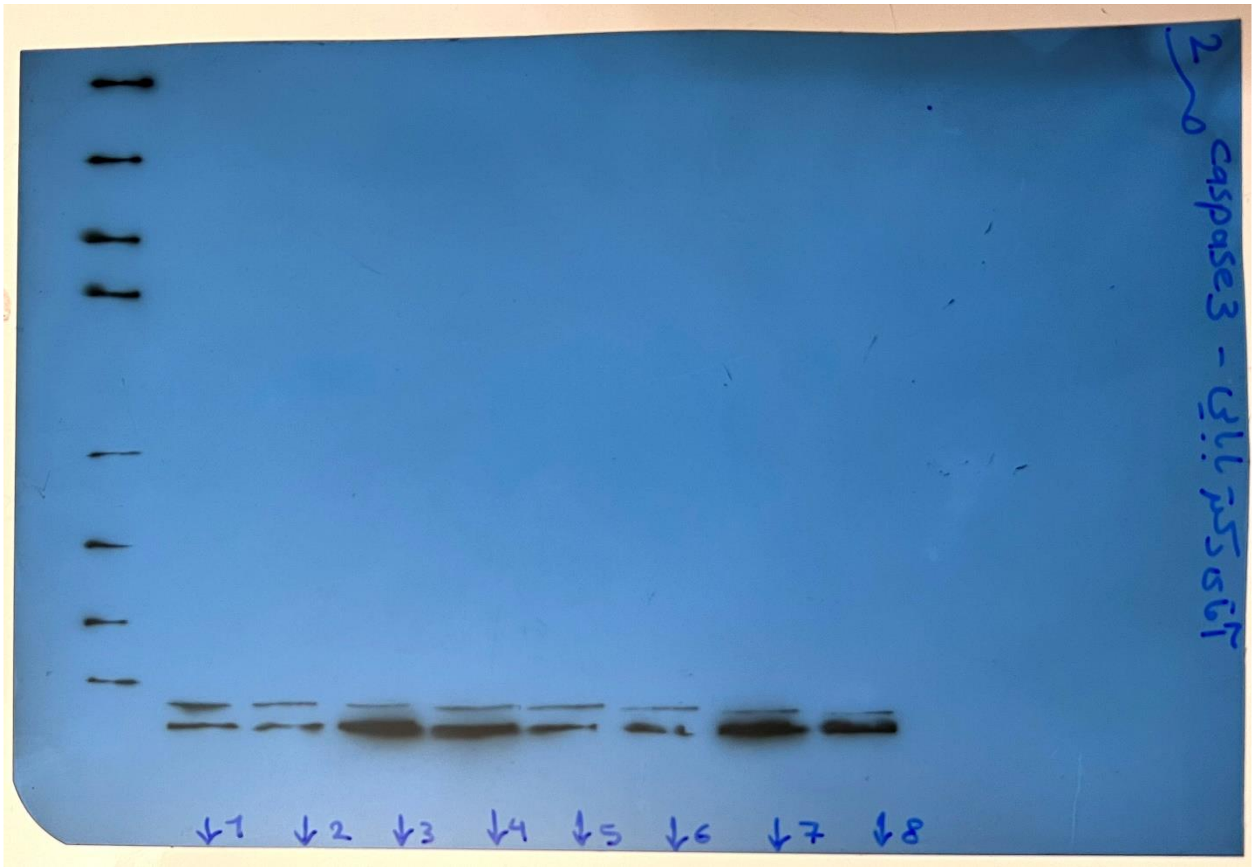

Fig S8: X-ray film of a Western blot showing Caspase-3 protein expression in hippocampal tissue (page 2).

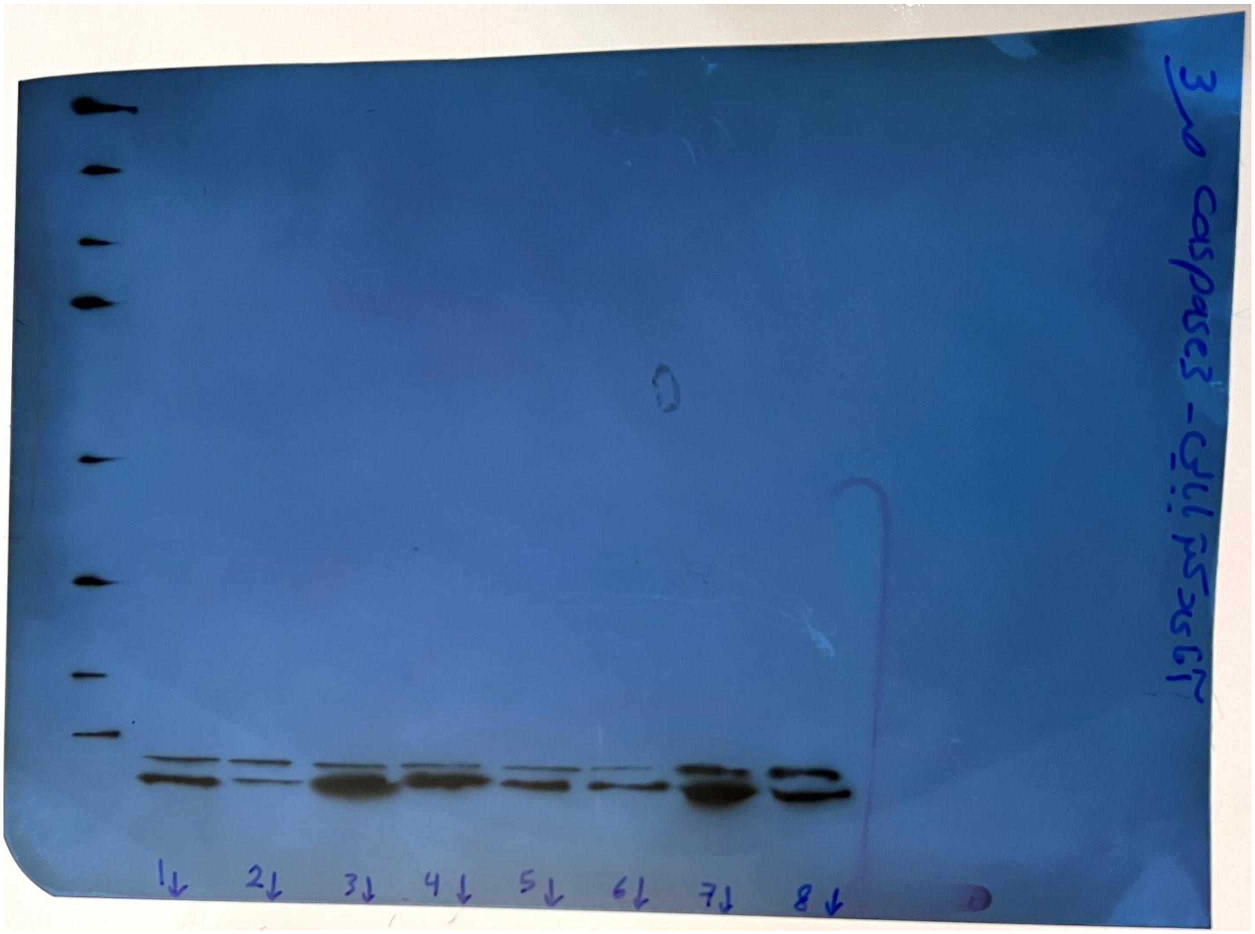

Fig S9: X-ray film of a Western blot showing Caspase-3 protein expression in hippocampal tissue (page 3).

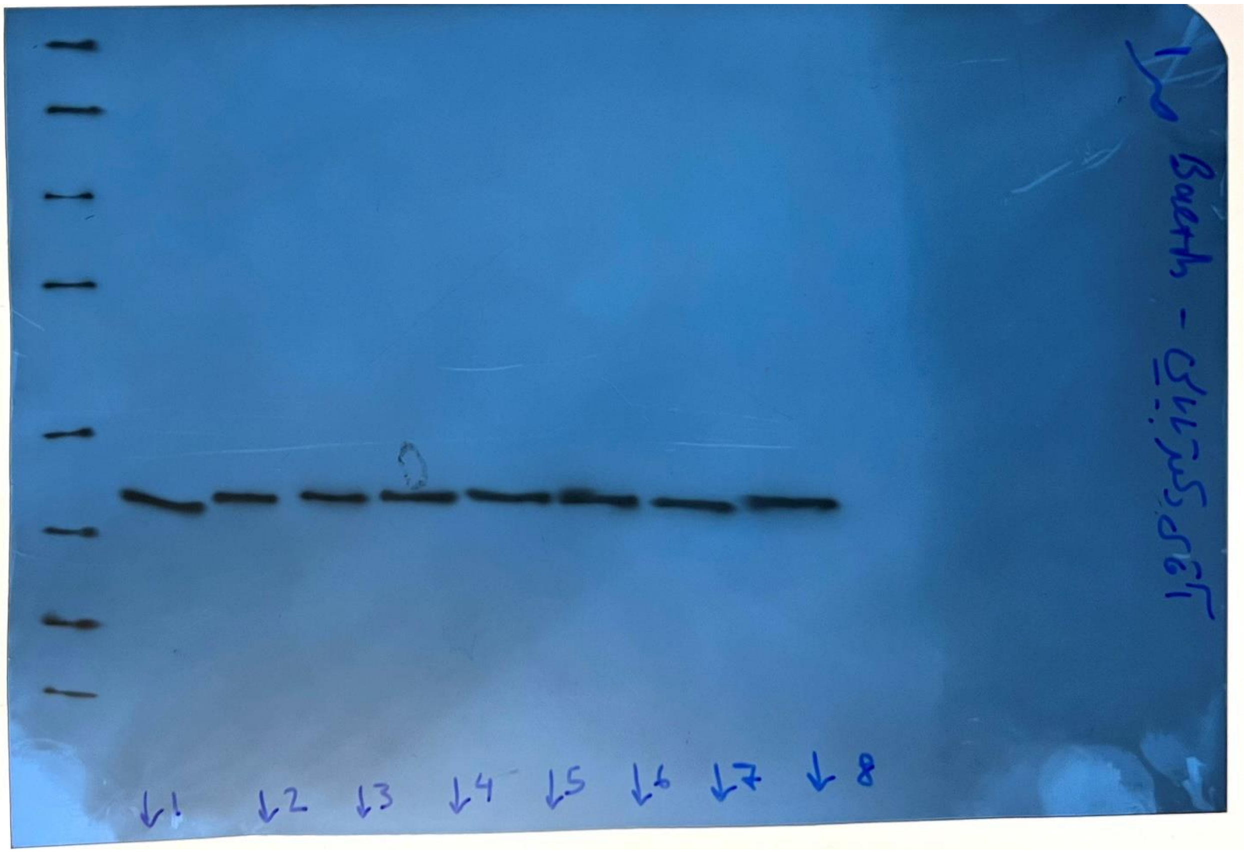

Fig S10: X-ray film of a Western blot showing β- Actin protein expression in hippocampal tissue (page 1).

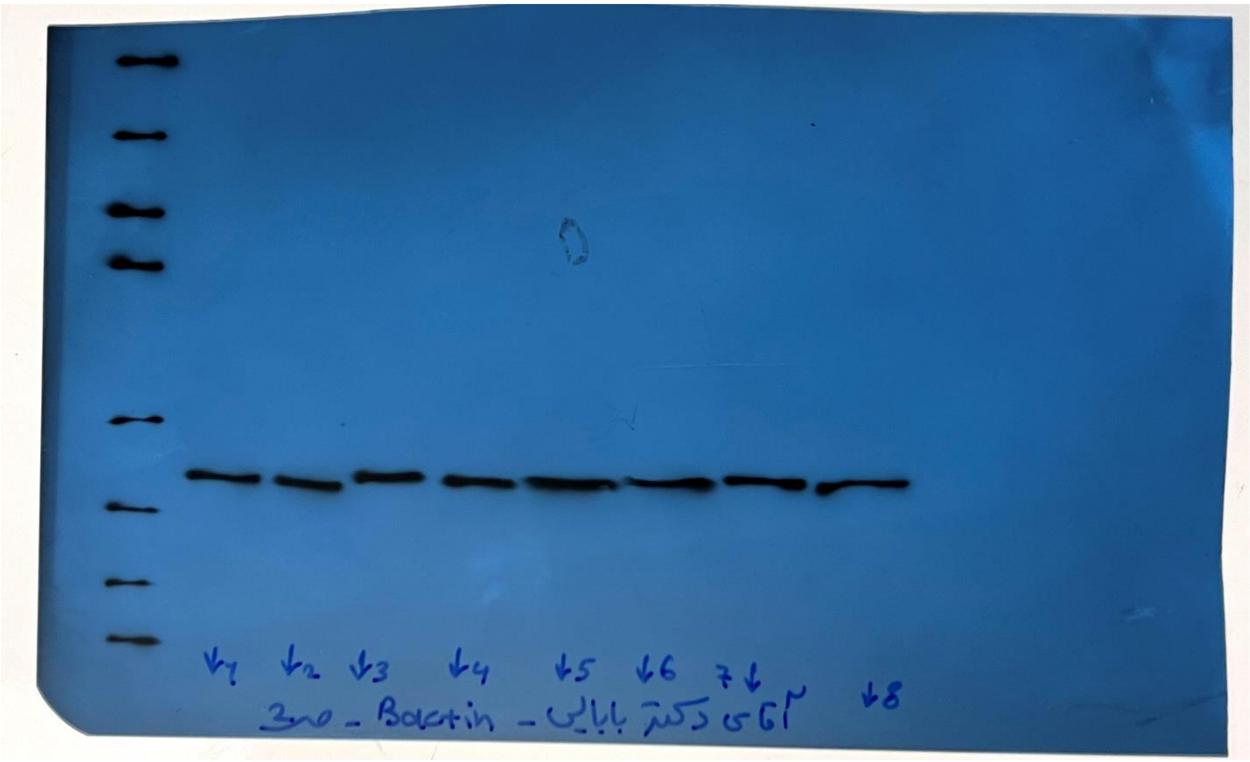

Fig S11: X-ray film of a Western blot showing β- Actin protein expression in hippocampal tissue (page 2).

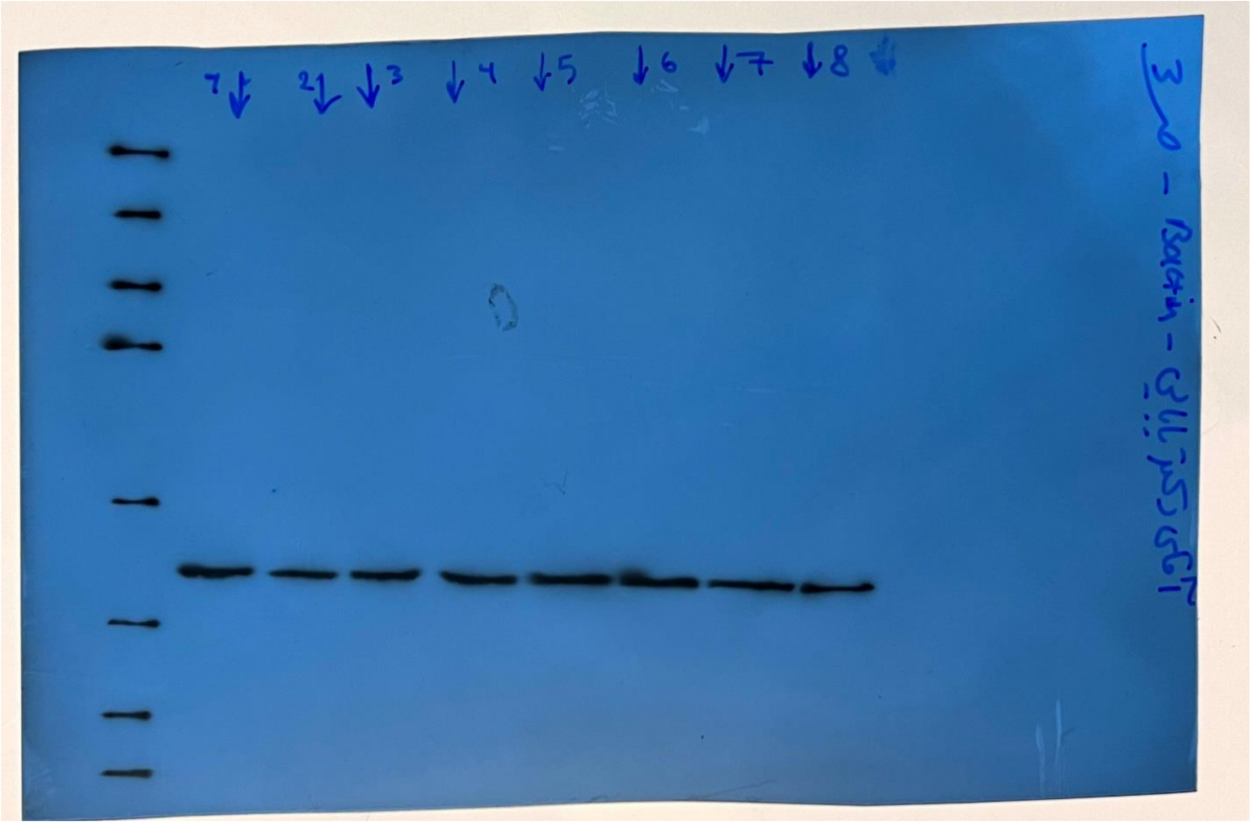

Fig S12: X-ray film of a Western blot showing β- Actin protein expression in hippocampal tissue (page 3).

Groups:

1[↓]: Male Control; 2[↓]: Male Sham Melatonin; 3[↓]: Male Sevoflurane; 4[↓]: Male Sevoflurane Melatonin

5[↓]: Female Control; 6[↓]: Female Sham Melatonin 7[↓]: Female Sevoflurane; 8[↓]: Female Sevoflurane Melatonin
